# Very low risk of monkeypox among staff and students after exposure to a confirmed case in educational settings, England, May to July 2022

**DOI:** 10.2807/1560-7917.ES.2022.27.40.2200734

**Published:** 2022-10-06

**Authors:** Shamez N Ladhani, Felicity Aiano, David S Edwards, Samantha Perkins, Wazirzada M Khan, Nalini Iyanger, Elizabeth Whittaker, Jonathan M Cohen, David Ho, Susan Hopkins, Mary E Ramsay, J Yimmy Chow

**Affiliations:** 1 Immunisation Division, UK Health Security Agency, London, United Kingdom; 2 Paediatric Infectious Diseases Research Group, St. George’s University of London, London, United Kingdom; 3 UK Health Security Agency East of England Health Protection Team, Mildenhall, United Kingdom; 4 UK Health Security Agency, South London Health Protection Team and the London Coordination and Response Cell, London, United Kingdom; 5 UK Health Security Agency Northwest London Health Protection Team and the London Coordination and Response Cell, London, United Kingdom; 6 Paediatric Infectious Diseases Department, Imperial College London, London, United Kingdom; 7 Paediatric Immunology and Infectious Diseases, Evelina London Children's Hospital, London, United Kingdom; 8 Clinical and Public Health Group, UK Health Security Agency, London, United Kingdom

**Keywords:** monkeypox, exposure, outbreak, school, vaccine

## Abstract

We investigated a secondary school (11–16 year-olds), a primary school (5–11 year-olds), reception year (4–5 year-olds) and a nursery (2–5 year-olds) following confirmed monkeypox in an adult in each educational setting during June and July 2022. MVA-BN vaccine was offered up to 14 days post exposure to 186 children < 12 years and 21 were vaccinated. No secondary cases occurred among at least 340 exposed students and more than 100 exposed staff during the 28-day follow-up period.

The current global monkeypox outbreak has by 13 September 2022 led to more than 60,000 confirmed or suspected cases in 114 countries [1], including 19,379 confirmed cases in 29 countries in the European Union and European Economic Area (EU/EEA) [2]. 

Despite the high numbers of confirmed monkeypox cases so far, there have been very few cases in children, who account for less than 1% of confirmed cases globally [3,4]. As children return to school after the summer holidays in many parts of the world, concerns have been raised about the risk of monkeypox in educational settings [5]. Here we report our experience with four incidents of exposure of children of different ages from confirmed adult monkeypox cases, which took place in different schools and nurseries during June and July 2022 in England.

## Monkeypox in England

In England, the first monkeypox case linked with the current outbreak was diagnosed on 6 May 2022. By 16 September 2022, there were 3,439 confirmed cases, with most cases initially identified in the London region [6]. The proportion of new cases which were in London has decreased over the course of the outbreak but has ranged between 50% and 70% since July 2022. Of the 3,390 cases with known gender, 99% were men and only 45 were women. Only one child aged 0–15 years has been diagnosed with monkeypox in England [6].

## Monkeypox exposure in educational settings

The national monkeypox incident team at the United Kingdom Health Security Agency (UKHSA) was involved with supporting four separate monkeypox exposures in educational settings during June and July 2022, including one secondary school (11–16 year-olds), one primary school (5–11 year-olds), one reception year of a primary school (4–5 year-olds and one nursery (2–5 year-olds) (Table). The index cases were all adult staff members who had spent considerable time on the school premises while symptomatic with a rash and had close contact with some students inside and/or outside the classroom before their diagnosis. Risk of exposure at the time of the study was categorised according to UK Health Security Agency guidance (Box) [7].

**Table:** Summary of monkeypox exposures in four educational settings in chronological order, England, June–July 2022

	Situation	Exposure period in educational setting	Case details	Exposure^a^	Management
1	Secondary school	5–17 May	Vesicles mainly on genital area, one lesion on wrist and ear, associated fever and feeling unwell	≥ 154 students (Cat 2/Cat 1), ≥ 100 staff (Cat 2)	Children excluded from school for 21 days from last exposure; Vaccination not offered because older children categorised as low risk of severe disease
2	Primary school	13–23 June	Muscle aches, fever, shivers, fatigue, dry throat, followed by rash a few days later, located on head, ears, chest, knee, leg and stomach	3 classes involving 38 children (Cat 2)^b^, 6 adults (5 Cat 2, 1 Cat 1)	Exposed children isolated from school for 21 days from last exposure; Vaccination not offered to adults as none were high-risk; Vaccine offered to 38 children; Vaccine taken by 10 children
3	Nursery	25 June–5 July	Started with blisters on tongue, followed 5 days later with fever headache, muscle pains and “swollen gland” which started at work. Rash developed on day 8, after exclusion from school.	58 children aged 2–3 years (Cat 2), 4 staff contacts (Cat 2)	Children excluded from school for 21 days from last exposure; Vaccination not offered to adults as none were high-risk; Vaccine offered to 58 children; Vaccine taken by 7 children
4	Reception year of a primary school (4–5 year-olds)	4–7 July	Fever and influenza-like symptoms, followed by vesicles on hands, arms and legs 2 days later, felt better after 3 days and was advised to return to work.	3 classes involving ca 90 children (Cat 2), 8 staff contacts (Cat 2), 2 parents who had a meeting with the case (Cat 2)	Children remained in school in class bubbles for 21 days or until end of term; One high-risk staff offered vaccine; Vaccine offered to all children (ca 90); Parents of 58 children indicated their interest in their child being vaccinated; Vaccine taken by 4 children

CAT: category.

^a^ Exposures were categorised in detail according to UK Health Security Agency guidance [7] – potential exposures related to educational settings included: Category 3 (high risk): direct exposure to broken skin or mucous membranes of case, their body fluids or potentially infectious material (including clothing); Category 2 (medium risk): contact within one metre with the case for at least 15 min; or, sharing a car with case; Category 1 (low risk): maintaining a distance of more than one metre from the case with no direct contact with case or their body fluids.

^b^ Two children were tested for suspected monkeypox because they had non-specific viral symptoms but were negative.

Box **Risk categories for monkeypox exposure**

- Category 3 (high risk): direct exposure to broken skin or mucous membranes of case, their body fluids or potentially infectious material (including clothing);
- Category 2 (medium risk): contact within 1 m with the case for at least 15 min; or, sharing a car with case;
- Category 1 (low risk): maintaining a distance of more than 1 m from the case with no direct contact with case or their body fluids.

UK Health Security Agency guidance [7].

For all incidents, public health action was initiated as soon as monkeypox diagnosis was confirmed in the index case with a positive RT-PCR at the UKHSA reference laboratory. Local UKHSA Health Protection Teams (HPTs) initiated contact tracing with the respective educational settings and identified staff and children who were potentially exposed to cases during their infectious periods. Daily incident management meetings were held, and exposed contacts were excluded from school for 21 days (but without the need for self-isolation at home) in the first three exposures settings and isolated into bubbles in the final exposure setting (reception year). Contacts were advised to avoid contact with high-risk individuals, such as other young children, pregnant women and immunosuppressed individuals, where possible. With the support of public health and paediatric colleagues, online webinars were also held to inform parents of exposed children and answer questions.

The first outbreak was a secondary school where at least 154 students and more than 100 staff were potentially exposed to a symptomatic adult staff member with confirmed monkeypox over a period of nearly 2 weeks. At the time, the UKHSA monkeypox guidance recommended MVA-BN (Modified Vaccinia Ankara - Bavarian Nordic) vaccination for high-risk close contacts (children aged < 12 years, pregnant women and immunocompromised individuals), ideally within 4 days of exposure (but could be given up to 14 days post exposure) to prevent secondary cases, with the aim of reducing the severity of any breakthrough infection [8]. Given that the majority of contacts between the case and exposed students and staff were assessed to be neither close or prolonged although most exposures were classified as Category 2, the time since exposure had exceeded 4 days – and more than 14 days for many of the contacts – and the risk of severe monkeypox in adolescents and adults is low, MVA-BN vaccine was not offered in this incident [8].

In the primary school incident, three classes involving 38 children aged 5–11 years and six staff members were exposed to a confirmed monkeypox case. Potential contacts were identified through public health investigations and excluded from school for 21 days. Given that the interval since exposure had exceeded 4 days, staff members were not offered vaccination, but all 38 children were offered one dose of MVA-BN vaccine, with 10 children eventually vaccinated within 14 days of last exposure.

In the nursery incident, the children and staff were exposed for a prolonged period in the educational setting, with confirmed physical contact between the case and some children (Category 2), since the young age of the children required them to be routinely carried and comforted by nursery staff. Public health investigations identified 58 children aged 2–3 years and four staff members as potential contacts – they were excluded from nursery for 21 days after last exposure. Here, too, staff members were not offered vaccination, but all 58 children were offered MVA-BN vaccine and seven were eventually vaccinated within 14 days of last exposure. 

In the final outbreak involving a reception year of a primary school, three classes involving 90 children aged 4–5 years, eight staff members and two parents who had a meeting with the confirmed case were potentially exposed. With increasing evidence of a very low risk of monkeypox in educational settings, close contacts with Category 2 exposure who were children were allowed to remain in school but in separate bubbles from the other unexposed children. One staff member at high risk of severe disease was given MVA-BN and all 90 potentially exposed children were offered one dose of MVA-BN, of whom four were eventually vaccinated within 14 days of last exposure.

In total, 186 children aged 2–11 years were offered one dose of MVA-BN vaccine and 21 (11%) were vaccinated, mostly between 7 and 14 days after exposure. Follow-up with local HPTs and the national UKHSA diagnostic laboratories did not identify a single case among at least 340 exposed students and more than 100 exposed staff members during the 28-day follow-up after the last exposure to the index cases in any of the four educational settings (Table).

## Discussion

The current global monkeypox outbreak mainly affects gay, bisexual and other men who have sex with men (GBMSM), with transmission occurring primarily through close physical, intimate and sexual contact. Transmission may, however, also occur through contaminated surfaces or droplets and, in individuals with respiratory tract symptoms or severe disease, the possibility of airborne transmission cannot be excluded [9]. While the majority of cases recover without complications, limited data suggest that pregnant women, immunocompromised individuals and young children may be at increased risk of monkeypox [10,11]. Before the current monkeypox outbreak, however, our understanding of monkeypox in children was limited to scanty reports from endemic countries and individual case reports from non-endemic countries [11,12], focussing mainly on the course of illness but not the risks from exposure to a confirmed case.

As monkeypox cases continue to increase globally, there is growing concern of spill-over of cases from GBMSM into the wider community. In England, for example, cases in women have gradually increased to 41 as per 26 August 2022, although, so far, only one child has been diagnosed with monkeypox [6]. Reassuringly, monkeypox cases in England have been declining since mid-July 2022, through a combination of public health messaging to avoid high-risk behaviour and seek early medical help, and a national immunisation programme for GBMSM, pre-exposure vaccination for healthcare workers and pre- and post-exposure vaccination for close contacts of confirmed cases.

So far, there have been no reports of monkeypox transmission in any educational setting in the current global outbreak [3,13], although a child was recently sent home with monkeypox in Oregon, United States, even though this child did not contract the virus at school, childcare or another community setting [14]. In our investigations, too, there were no secondary cases among students or staff despite prolonged exposure to an adult with confirmed monkeypox. The secondary school outbreak involved potential exposure to a large number of staff and students, but the public health assessment identified most exposures to be of low risk because they did not involve close or prolonged contact with the case. Since many of the exposures had exceeded 14 days by the time public health investigations were initiated, it was already reassuring that there were no symptomatic or suspected cases among exposed contacts. In contrast to the secondary school, close contact between staff and the students was more likely to occur with the younger children in primary school, in the reception year and, almost inevitably, in the nursery. While the same public health actions were implemented in these three settings, vaccination was prioritised for exposed children and high-risk adults, although vaccine uptake among children was very low and invariably outside the recommended 4-day post-exposure period. Consequently, post-exposure vaccination is unlikely to have contributed to the observed low risk of secondary monkeypox among exposed contacts. In addition, the UK risk categories do not differentiate between skin-to-skin contact and respiratory contact, which is the most likely exposure for most contacts in educational settings, and respiratory exposure is likely to be a very low risk for monkeypox compared with skin-to-skin contact [7].

The current global monkeypox outbreak is caused by Clade IIb (formerly West African clade) of the virus which is usually associated with milder disease than the Central African clade [6]. In England, as more evidence became available during the course of the national monkeypox outbreak, it became apparent that Category 2 (medium-risk) and Category 1 (low-risk) exposures were associated with a very low risk of developing the disease [6]. As a result, public health advice was updated on 25 July 2022, such that close contacts of a confirmed case were no longer required to isolate. This included contacts who are children with any level of exposure who were no longer excluded from school unless symptomatic – this approach should significantly reduce the resources and time spent on extensive contact tracing for future incidents, unless additional cases are identified in the same setting [7]. Exposed contacts should, however, be informed to self-isolate and seek medical advice if they become symptomatic. Moreover, Category 3 (high-risk) contacts, are advised to avoid contact with children younger than 5 years, pregnant women and severely immunosuppressed individuals, where possible. In addition, the standard advice to avoid sexual or intimate contact with others for a full 21 days still applies. Currently, MVA-BN vaccination is recommended for Category 3 (high-risk) and Category 2 (medium-risk) exposures, ideally within 4 days of exposure, but up to 14 days since last exposure if there is a high risk of ongoing exposure or if contacts are immunosuppressed, pregnant women or children under 5 years of age.

## Conclusions

The low risk of developing monkeypox among staff and students after exposure to a confirmed case in educational settings is reassuring. The risk of transmission from infected children to other children or adults, however, is currently unknown. Given that nearly all childhood monkeypox cases have been acquired through household transmission, public health actions need to focus on contact tracing for all confirmed cases, offer vaccination where eligible and advise to isolate and seek medical advice if symptomatic. Current public health messaging for adults on risks, prevention and seeking medical help, including the offer of vaccination, should be extended to adolescents.
